# Complete plastome sequence of *Ilex asprella* (Hooker and Arnott) Champion ex Bentham (Aquifoliaceae), a Chinese folk herbal medicine

**DOI:** 10.1080/23802359.2019.1629347

**Published:** 2019-07-12

**Authors:** Yan Chen, Hua-Xu Chen, Hai-Li Li, Xia-Lan Cheng, Li-Yun Wang

**Affiliations:** Lingnan Normal University, Zhanjiang, China

**Keywords:** *Ilex asprella*, plastome, phylogeny, genome structure, Aquifoliaceae

## Abstract

The complete chloroplast genome of *Ilex asprella*, a species of Aquifoliaceae is reported for the first time in this study. The complete chloroplast genome of *I. asprella* is 157,856 bp in length with a typical quadripartite structure, consisting of a large single-copy region (LSC, 87,258 bp), a single-copy region (SSC, 18,441 bp) and a pair of inverted repeats (IRs, 26,082 bp). There are 114 genes annotated, including 85 unique protein-coding genes, four unique ribosomal RNA genes, and 30 transfer RNA genes. To investigate the evolution status of *T. concolor*, as well as Scrophulariaceae, we build a phylogenetic tree with *I. asprella* and other eight species based on their complete chloroplast genomes. According to the phylogenetic topologies, *I. asprella* was closely related to *I. wilsonii*.

*Ilex asprella* (Hooker & Arnott) Champion ex Bentham belongs to the family Aquifoliaceae. It produces and stores a large amount of triterpenoid saponins and is widely used as a folk herbal drug in southern China (Zheng et al. 2015). *Ilex asprella* distributes mainly in eastern and southern provinces of China, Philippines, and Vietnam (China ECoFo, 2013). Chloroplast is important in phylogeny reconstruction due to carrying maternal genes. Since there is no published plastome sequences data for *I. asprella* chloroplast in the present, the genetic and genomic information is urgently needed to promote its systematics research. We report and characterize the complete plastid genome sequence of *I. asprella* (GenBank accession number: MK834323) in an effort to provide genomic resources useful for promoting its conservation and utilization.

In this study, the fresh leaves of *I. asprella* were collected from its natural habitat Pangkan village, Suixi county, China (E110°18′40.45″N21°20′07.45″). Voucher specimens (LNH180925042) were deposited in the Herbarium of Lingnan Normal University, Zhanjiang, China. The experiment procedure is as reported in Gao et al. (2017). Around 6 Gb clean data were assembled against the plastome of *I. wilsonii* (KX426471.1) using MITO bim V1.8 (Hahn et al. 2013). The plastome was annotated using Geneious R8.0.2 (Biomatters Ltd., Auckland, New Zealand) against the plastome of *I. wilsonii* (KX426471.1). The annotation was corrected with DOGMA (Wyman et al. 2004).

The plastome of *I. asprella* was found to possess a total length 157,856 bp with the typical quadripartite structure of angiosperms, containing two Inverted Repeats (IRs) of 26,082 bp, a Large Single-Copy (LSC) region of 87,258 bp and a Small Single-Copy (SSC) region of 18,441 bp. The plastome contains 114 genes, consisting of 85 unique protein-coding genes, 30 unique tRNA genes, and four unique rRNA genes. The overall A/T content in the plastome of *I. asprella* is 62.40%, and the corresponding values for the LSC, SSC, and IR regions were 64.40%, 68.10%, and 57.00%, respectively.

We used RAxML (Stamatakis, 2006) with 1000 bootstraps under the GTRGAMMAI substitution model to reconstruct a maximum likelihood (ML) phylogeny of *I. asprella* and eight published complete plastomes, using *Lobelia erinus* and *Lobelia baumannii* as outgroups. According to the phylogenetic topologies, *I. asprella* was closely related to *I. wilsonii*. Most nodes in the plastome ML trees were strongly supported (Figure 1). The complete plastome sequence of *I. asprella* will provide a useful resource for the conservation genetics of this species as well as for building the phylogenetic relationships of Aquifoliaceae.

**Figure 1.**
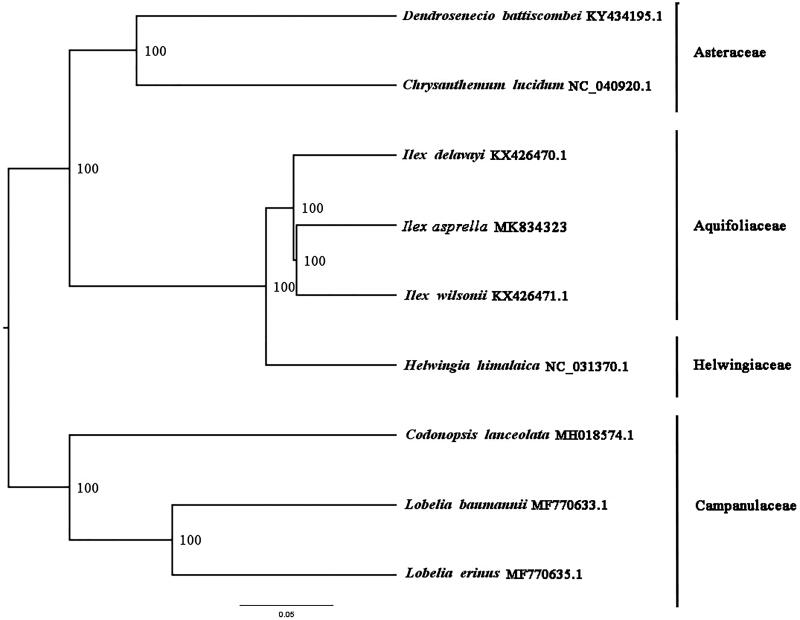
ML phylogenetic tree of *I. wilsonii* with nine species was constructed using chloroplast genome sequences. Accession numbers: *Chrysanthemum lucidum* NC_040920.1, *Dendrosenecio battiscombei* KY434195.1, *Ilex asprella* MK834323, *Ilex delavayi* KX426470.1, *Ilex wilsonii* KX426471.1, *Helwingia himalaica* NC_031370.1, *Codonopsis lanceolata* MH018574.1, *Lobelia banmannii* MF770633.1, *Lobelia erinus* MF770635.1. *Lobelia erinus* and *Lobelia baumannii* were selected as outgroups.
